# Immediate Post-operative Enterocyte Injury, as Determined by Increased Circulating Intestinal Fatty Acid Binding Protein, Is Associated With Subsequent Development of Necrotizing Enterocolitis After Infant Cardiothoracic Surgery

**DOI:** 10.3389/fped.2020.00267

**Published:** 2020-05-27

**Authors:** John D. Watson, Tracy T. Urban, Suhong S. Tong, Jeanne Zenge, Ludmilla Khailova, Paul E. Wischmeyer, Jesse A. Davidson

**Affiliations:** ^1^Department of Pediatrics, University of Colorado, Aurora, CO, United States; ^2^Research Institute, Children's Hospital Colorado, Aurora, CO, United States; ^3^Department of Biostatistics, Children's Hospital Colorado/University of Colorado, Aurora, CO, United States; ^4^Duke University Department of Anesthesiology, Durham, NC, United States

**Keywords:** biomarker, pediatric, congenital heart disease, IFABP, post-operative care, nutrition, NEC, cardiopulmonary bypass

## Abstract

**Objectives:** 1 Measure serial serum intestinal fatty acid binding protein levels in infants undergoing cardiac surgery with cardiopulmonary bypass to evaluate for evidence of early post-operative enterocyte injury. 2 Determine the association between immediate post-operative circulating intestinal fatty acid binding protein levels and subsequent development of necrotizing enterocolitis.

**Design:** Observational cohort study. Intestinal fatty acid binding protein was measured pre-operatively, at rewarming, and at 6 and 24 h post-operatively. Percent of goal enteral kilocalories on post-operative day 5 and episodes of necrotizing enterocolitis were determined. Multivariable analysis assessed for factors independently associated with clinical feeding outcomes and suspected/definite necrotizing enterocolitis.

**Setting:** Quaternary free-standing children's hospital pediatric cardiac intensive care unit.

**Patients:** 103 infants <120 days of age undergoing cardiothoracic surgery with cardiopulmonary bypass.

**Interventions:** None.

**Results:** Median pre-operative intestinal fatty acid binding protein level was 3.93 ng/ml (range 0.24–51.32). Intestinal fatty acid binding protein levels rose significantly at rewarming (6.35 ng/ml; range 0.54–56.97; *p* = 0.008), continued to rise slightly by 6 h (6.57 ng/ml; range 0.75–112.04; *p* = 0.016), then decreased by 24 h (2.79 ng/ml; range 0.03–81.74; *p* < 0.0001). Sixteen subjects (15.7%) developed modified Bell criteria Stage 1 necrotizing enterocolitis and 9 subjects (8.8%) developed Stage 2 necrotizing enterocolitis. Infants who developed necrotizing enterocolitis demonstrated a significantly higher distribution of intestinal fatty acid binding protein levels at both 6 h (*p* = 0.005) and 24 h (*p* = 0.005) post-operatively. On multivariable analysis, intestinal fatty acid binding protein was not associated with percentage of goal enteral kilocalories delivered on post-operative day 5. Higher intestinal fatty acid binding protein was independently associated with subsequent development of suspected/definite necrotizing enterocolitis (4% increase in odds of developing necrotizing enterocolitis for each unit increase in intestinal fatty acid binding protein; *p* = 0.0015).

**Conclusions:** Intestinal fatty acid binding protein levels rise following infant cardiopulmonary bypass, indicating early post-operative enterocyte injury. Intestinal fatty acid binding protein was not associated with percent of goal enteral nutrition achieved on post-operative day 5, likely due to protocolized feeding advancement based on clinically observable factors. Higher intestinal fatty acid binding protein at 6 h post-operatively was independently associated with subsequent development of necrotizing enterocolitis and may help identify patients at risk for this important complication.

## Introduction

Infants undergoing surgery for congenital heart disease (CHD) with cardiopulmonary bypass (CPB) are at high risk for intestinal injury, both during the surgery itself and subsequently from ongoing post-operative low cardiac output (1, 2). This intestinal injury can lead to major postoperative complications including intestinal barrier dysfunction, dysmotility, post-operative feeding intolerance, and post-operative necrotizing enterocolitis (NEC) (3–13). NEC, in particular, is a dangerous complication. Comorbid CHD and NEC have been shown to have high overall mortality (50–57%) (14, 15) and significantly increased length of hospitalization compared to CHD alone (36 vs. 19 days) (16). At this time, no clinically available biomarker exists to help identify specific intestinal damage following infant cardiac surgery.

Intestinal epithelial injury can be assessed by minimally invasive testing of intestinal fatty acid-binding protein (IFABP). IFABP is a 15-kD cytosolic protein localized mainly to mature enterocytes of the small intestinal villi with normally low circulating levels (17–20). IFABP plasma concentration has been shown to correlate to intestinal epithelial injury in animal models (21, 22). Much of the recent research involving IFABP has been as a predictor of NEC in the neonatal population (23–25), but it has also been utilized as an indicator of intestinal injury across many different contexts including cardiac surgery in both adults and children (3, 26–30). Previous studies performed in mixed age cohorts of children undergoing surgery for CHD indicate that IFABP levels rise immediately following surgery and then fall to below preoperative levels in the subsequent recovery period (3, 26). However, no previous studies have looked at IFABP levels in specifically the *infant* CHD population, which is a unique and important age group given the complexity of the surgeries performed at this age and the risk for diffuse organ injury. Additionally, associations between IFABP levels and feeding tolerance or clinical suspicion of NEC have not been assessed in this high-risk population.

In this study, we sought to describe the pattern of serum IFABP in infants undergoing CPB. In addition, we aimed to determine the clinical risk factors associated with elevated preoperative and postoperative IFABP levels, hypothesizing that higher IFABP levels would be associated with worse clinical severity of disease. Finally, we investigated whether there was an association between IFABP and early enteral feeding outcomes/development of NEC, hypothesizing that greater elevation of IFABP levels in the immediate post-operative period would predict worse enteral feeding outcomes and greater odds of developing NEC.

## Materials and Methods

This study was a pre-specified secondary aim of a prospective cohort study evaluating alkaline phosphatase activity after infant CPB (31), with prospective collection of samples for IFABP analysis and *post-hoc* analysis of infant feeding outcomes. Over a period of two-and-a-half years from September 2013 to February 2016, infants ≤ 120 days at time of cardiothoracic surgery with use of CPB were enrolled. Exclusion criteria were weight <2 kg (as limited blood volume could increase risk of anemia with research blood draws), adjusted gestation age <34 weeks (given concerns for altered biomarker production), and prior participation in this protocol. The protocol was approved by the Colorado Multiple Institution Review Board, and informed consent was obtained from the subjects' parents prior to enrollment. The primary aims of the current study were to measure pre- and post-op IFABP levels in an infant cohort undergoing CPB and determine the association between IFABP levels and early enteral feeding outcomes and development of NEC.

### Operative and Post-operative Management

As previously published (32), CPB was performed using a neonatal circuit consisting of a roller head pump (S5, LivaNova, Arvada, CO, USA) and a Terumo FX05 oxygenator with a blood prime. The blood prime routinely underwent pre-bypass hemofiltration using a Minntech Hemocor HPH Junior hemoconcentrator (Medivators Inc., Minneapolis, MN, USA) with a polysulfone membrane prior to initiating bypass, allowing for partial filtration of molecules up to 65,000 Daltons. Anticoagulation was achieved prior to CPB by administering 500 units/kg of heparin systemically to the patient. Initial target flow rate was ~200 ml/kg/minute. Cardioplegia was accomplished using del Nido formula cardioplegia solution at an initial dose of 30 ml/kg and subsequent dosing was considered after 60 min of aortic cross-clamp time. Per our clinical protocol, all neonates (age <1 month) received high dose methylprednisolone (10 mg/kg) at 10 and 4 h prior to surgery to attempt to reduce post-CPB systemic inflammation.

### Sample Collection and Analysis

IFABP levels were obtained pre-operation, immediately prior to withdrawal of CPB, and 6 and 24-h post-operation. Serum concentration of IFABP was analyzed at the University of Colorado, Denver using Meso Scale Discovery (MSD) multiplex immunoassay system, Meso Scale Diagnostics, LLC, Gaithersburg, MD.

### Clinical Variables

Pre-operative, intraoperative, and post-operative variables were collected to evaluate for potential risk factors for pre- and post-operative intestinal damage. Pre-operative variables included age, weight, sex, Aristotle score (comprehensive and basic) (33), pre-operative mechanical ventilation, pre-operative inotropic support, pre-operative initiation of enteral feeding, prostaglandin use, single ventricle physiology, and prematurity. Intraoperative and post-operative variables included CPB time, cross-clamp time, deep hypothermic circulatory arrest time, selective cerebral perfusion time, duration of mechanical ventilation, vasoactive inotropic score (VIS) (34, 35) at 6-h post-operative, and clinically obtained peak creatinine and peak lactate levels.

### Post-operative NEC

Diagnosis and staging of NEC was made based on the modified Bell's Staging Criteria via retrospective chart review (36). Although the Bell Staging Criteria are designed primarily for NEC associated with prematurity, they remain the primary diagnostic criteria for cardiac-associated NEC (16, 37–39). Only post-operative cases of NEC during the same hospitalization as the operation were included.

### Infant Feeding Outcomes

A *post-hoc* data collection from the electronic medical record was utilized to gather nutrition and feeding data. We examined the primary feeding outcome of percent of goal feeds achieved enterally on post-op day 5. Additionally, we examined secondary outcomes of percent of goal feeds achieved on post-op day 3. To calculate the percent of goal feeds achieved enterally, we took the patient's achieved enteral feeds (in kcals) as recorded in the patient's electronic medical record flowsheet and divided this by the goal kcals as documented daily by the cardiac intensive care unit dieticians. The feeding protocol utilized in our CICU is included as Supplemental Figure 1.

### Statistical Analysis

Patients' demographics and clinical characteristics are summarized using descriptive statistics. The comparison groups were divided by IFABP level below and equal or above 50th percentile at the given time point. The distributions of continuous variables were inspected before data analysis, all the continuous variables were summarized with median and range (min and max). Wilcoxon rank sum test was conducted to determine the significant difference in distributions between the comparison groups. The categorical data was summarized as count and percentage for the “Yes” category for a giving measurement. To model the association between percentage of goal enteral kilocalories achieved and IFABP, multivariable general linear model was used with pre-selected clinical variables as covariates. Backwards model selection strategy was used, and R-square and type III *P*-values were used to evaluate the most harmonious model. Multivariable logistic regression model was performed to assess the association between NEC and IFABP, demographic and clinical covariates were selected through univariate logistic regression with area under curve (AUC) >0.6, then added to the multivariable model. Akaike information criterion *(*AIC*)* was used to evaluate the best fitting model. *P*-values < 0.05 were considered as statistically significant. All statistical analysis was performed using SAS version 9.4 (SAS Institute, Cary, NC) and all plots were produced with GraphPad Prism version 8.

### Data Availability Statement

The raw data supporting the conclusions of this manuscript will be made available by the authors, without undue reservation, to any qualified researcher.

## Results

### Subjects

The study consecutively enrolled 100 and two subjects, plus one screen failure (converted to non-bypass surgery after enrollment). Of these subjects, one patient did not have a preoperative IFABP level, one patient did not have an IFABP level at rewarming, three patients did not have a 6 h IFABP level, and two patients did not have a 24 h IFABP level due to insufficient sample volume, leaving a total of 101 preoperative and rewarming samples, 99 six hour samples, and 100 twenty-four hour samples (99% overall collection rate). Table 1 lists the cardiac diagnoses and the operations performed. Clinical characteristics for this cohort are shown in Table 2.

**Table 1:** Diagnoses and operations performed.

**Diagnoses**	***n***	**%**	**Operation**	***n***	**%**
Hypoplastic left heart syndrome	20	19.6	Norwood procedure	19	18.6
VSD/aortic arch hypoplasia	13	12.7	VSD repair	14	13.7
Tetralogy of fallot	12	11.8	Tetralogy of fallot repair	12	11.8
Ventricular septal defect	11	10.8	Arterial switch	9	8.8
Atrioventricular septal defect	9	8.8	Aortic arch repair	8	7.8
Double outlet right ventricle	8	7.8	Atrioventricular septal defect repair	6	5.9
PA/VSD	5	4.9	TAPVR repair	5	4.9
TAPVR	5	4.9	Truncus arteriosus repair	5	4.9
Transposition of great arteries	5	4.9	Systemic-pulmonary shunt	4	3.9
Truncus arteriosus	4	3.9	VSD/aortic arch repair	4	3.9
Aortic arch hypoplasia	1	1.0	Double outlet right ventricle repair	3	2.9
Double inlet left ventricle	1	1.0	Glenn, pulmonary arterioplasty	2	2.0
PA/IVS	1	1.0	Mitral valve repair	2	2.0
Other	7	6.9	Right ventricle-pulmonary artery conduit	2	2.0
			Right ventricular outlet tract repair	2	2.0
			Cardiac tumor resection	1	1.0
			Left ventricular assist device	1	1.0
			Reimplantation of LPA from Ao to MPA	1	1.0
			Ross procedure	1	1.0
			Yasui procedure	1	1.0

*VSD, ventricular septal defect; PA, pulmonary atresia; TAPVR, total anomalous pulmonary venous return; IVS, intact ventricular septum; LPA, left pulmonary artery; Ao, aorta; MPA, main pulmonary artery*.

**Table 2:** Baseline clinical characteristics (described in full cohort column) and comparison of clinical characteristics between infants with lower (<50th percentile) and higher (>50th percentile) pre-operative IFABP levels.

**Baseline characteristics**	**Full cohort**	**Pre-operative IFABP level ≤50%**	**Pre-operative IFABP level >50%**	***p*-value ≤50 vs. >50**
Pre-operative IFABP level (ng/ml), median (range)	3.9 (0.2, 51.3)	1.9 (0.2, 3.9)	7.8 (4.3, 51.3)	**<0.0001**
Age at surgery, days; median (range)	21.5 (1, 120)	6.0 (1, 119)	55 (2, 120)	**0.0004**
Weight; median (range)	3.6 (2.1, 7.4)	3.3 (2.2, 7.4)	3.8 (2.1, 7.2)	**0.0474**
Birthweight; median (range)	3.1 (1.3, 4.6)	3.1 (2.3, 4.0)	3.0 (1.3, 4.6)	0.1598
Male (%)	57 (55.3%)	31 (60.8%)	25 (50%)	0.2756
Aristotle Score-Comprehensive; median (range)	9.0 (3.0, 19.5)	10 (3.0, 19.5)	9 (3.0, 15.0)	0.3177
Aristotle Score-Basic; Median (range)	9.0 (3.0, 15.0)	9.0 (3.0, 14.5)	8.5 (3.0, 15.0)	0.4033
Pre-operative mechanical ventilation (%)	32 (31.4%)	15 (29.4%)	16 (32%)	0.7780
Pre-operative inotropic support (%)	13 (12.6%)	9 (17.7%)	4 (8%)	0.2343
Single ventricle physiology (%)	29 (28.7%)	16 (32%)	12 (24%)	0.3730
Pre-operative enteral nutrition initiated (%)	95 (92.2%)	44 (86.3%)	50 (100%)	**0.0125**
Preterm (%)	15 (14.7%)	4 (7.8%)	11 (22.5%)	0.0518
Prostaglandins (%)	51 (50%)	35 (68.6%)	15 (30%)	**0.0001**

*Bold values represent statistically significant values (p < 0.05)*.

### IFABP Measurements and Association With Clinical Variables

**Figure 1:**
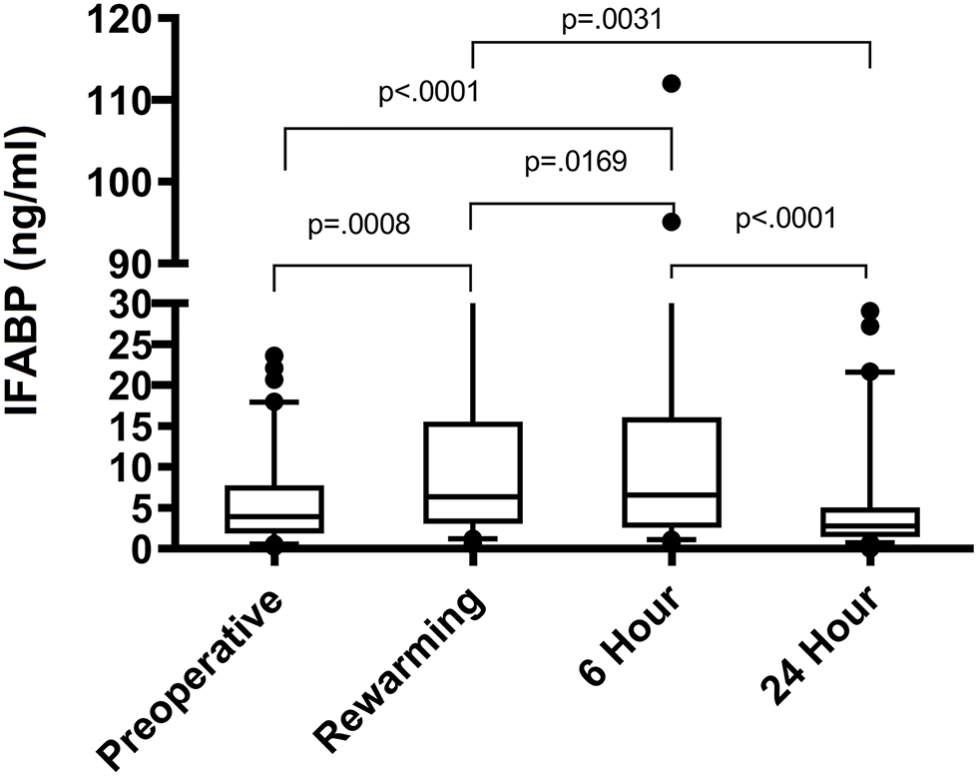
IFABP levels (ng/ml) shown over time at 4 different time points: pre-operative, rewarming, 6 h post-operative, and 24 h post-operative.

The distribution of IFABP levels initially rose at rewarming, increased slightly at 6 h, and by 24 h had trended down to near preoperative levels as shown in Figure 1. The association between clinical characteristics and IFABP levels pre-operatively are shown in Table 2. Pre-operative clinical characteristics found to be significantly associated with higher IFABP include older age, higher weight, prior initiation of pre-operative enteral nutrition, and lack of prostaglandin use. The association of operative and post-operative variables and IFABP levels at 6 h and at 24 h are shown in Table 3. The only variable found to be significantly associated with higher IFABP at 6-h post-operative was higher vasopressor-inotropic score (VIS). At 24 h, single ventricle physiology, longer CPB time, longer duration of mechanical ventilation, higher VIS, and higher peak creatinine were all found to be significantly associated with higher IFABP.

**Table 3:** Comparison of baseline, intra-operative, and post-operative characteristics between infants with lower (<50th percentile) and higher (>50th percentile) post-operative IFABP levels at 6 and 24 h.

**Baseline, intraoperative, and post-operative characteristics**	**IFABP level 6 h ≤50%**	**IFABP level 6 h >50%**	***p*-value ≤50 vs. >50**	**IFABP level 24 h ≤50%**	**IFABP level 24 h >50%**	***p*-value ≤50 vs. >50**
Pre-operative IFABP level (ng/ml); median (range)	2.7 (0.2, 16.1)	5.1 (0.6, 51.3)	**0.0261**	3.2 (0.4, 11.5)	5.1 (0.2, 51.3)	**0.0245**
Age at surgery, days; median (range)	14.0 (2, 119)	22 (1, 120)	0.5812	19.5 (2.0, 120.0)	28 (1, 115)	0.1975
Weight; median (range)	3.6 (2.2, 6.0)	3.5 (2.1, 7.4)	0.3542	3.6 (2.2, 7.4)	3.6 (2.1, 6.4)	0.5409
Birthweight; median (range)	3.2 (1.9, 4.3)	2.9 (1.3, 4.6)	0.1294	3.0 (1.7, 4.0)	3.1 (1.3, 4.6)	0.2061
Male (%)	28 (56%)	29 (59%)	0.7486	26 (52%)	29 (58%)	0.5465
Preterm (%)	5 (10%)	10 (20.8%)	0.1670	7 (14%)	8 (16.3%)	0.7469
Aristotle Score-Comprehensive; median (range)	10 (3,16)	9(3, 19.5)	0.4859	9 (3,16)	10 (3, 19.5)	0.3520
Aristotle Score-Basic; Median (range)	9 (3, 14.5)	8 (3,15)	0.5954	9 (3, 14.5)	9 (3, 15)	0.2234
Single ventricle physiology (%)	12 (24%)	17 (35%)	0.2158	8 (16%)	20 (40.8%)	**0.0061**
Pre-operative enteral nutrition initiated (%)	46 (92%)	46 (94%)	1.0000	47 (94%)	46 (92%)	0.6951
CPB time, minutes; median (range)	123.5 (54, 399)	131.0 (55, 372)	0.4726	119 (54, 399)	147.5 (55, 298)	**0.0259**
Cross-clamp time, minutes; median (range)	71 (0, 205)	69 (0, 241)	0.8642	66.5 (0, 241)	75 (0, 205)	0.2637
Deep hypothermic circulatory arrest, minutes; median (range)	0 (0, 77)	0 (0, 59)	0.8675	0 (0, 77)	0 (0, 76)	0.9145
Selective cerebral perfusion, minutes; median (range)	0 (0, 82)	0 (0, 115)	0.2516	0 (0, 82)	0 (0, 115)	0.0736
Duration of mechanical ventilation, hours; median (range)	32.5 (0.1, 213.7)	45.3 (0.5, 762.6)	0.1812	26.6 (0.1, 762.6)	51.7 (7.5, 237.8)	**0.0116**
VIS at 6h; median (range)	8 (2.5, 17)	10 (0, 27)	**0.0326**	5 (0, 18)	8.8 (0, 30)	**0.0055**
Lactate peak; median (range)	3.2 (1.1, 11.4)	3.4 (0.9, 12.6)	0.6806	3.2 (0.9, 12.6)	3.6 (1.1, 11.4)	0.2003
Peak Creatinine; median (range)	0.5 (1.3, 1.7)	0.5 (0.3, 1.9)	0.8972	0.5 (0.3, 1.1)	0.5 (0.3, 1.9)	**0.0347**
IL-6 at 6 h; median (range)	48.6 (10.2, 524.8)	57.0 (1.3, 296.9)	0.6327	43.5 (11.0, 351.0)	60.7 (12.3, 908.7)	0.1040
TNFα at 6 h; median (range)	7.6 (2.7, 24.7)	7.4 (3.1, 72.3)	0.8587	6.5 (3.4, 11.6)	6.8 (2.7, 29.2)	0.6183

*Bold values represent statistically significant values (p < 0.05)*.

### Post-operative NEC and Association With Clinical Variables

The median number of days from surgery to NEC onset was 10 days (IQR of 4–14.5; range 1–52). The association between clinical characteristics and post-operative NEC are shown in Table 4. Clinical characteristics found to be significantly associated with post-operative NEC include lower weight, longer deep hypothermic circulatory arrest time, longer duration of mechanical ventilation, higher VIS, higher peak lactate, and higher peak creatinine.

**Table 4:** Comparison of baseline, intra-operative, and post-operative characteristics between infants without NEC or with NEC as defined by bell stage 1 or Stage 2.

**Baseline, intraoperative, and post-operative characteristics**	**No NEC (*n* = 75)**	**NEC (Bell stage 1 or Stage 2) (*n* = 25)**	***p*-value**
Pre-operative IFABP level (ng/ml); median (range)	4.3 (0.2, 22.1)	3.4 (0.5, 51.3)	0.3844
Age at surgery, days; median (range)	36 (1, 120)	9 (2, 115)	0.1178
Weight; median (range)	3.7 (2.1, 7.4)	3.2 (2.3, 4.7)	**0.0040**
Birthweight; median (range)	3.2 (1.9, 4.6)	2.9 (1.3, 3.9)	0.1327
Male (%)	38 (50.7%)	18 (72%)	0.0627
Preterm (%)	9 (12%)	5 (20.8%)	0.3176
Aristotle score-comprehensive; median (range)	9 (3,16)	10 (3, 19.5)	0.4295
Aristotle score-basic; median (range)	9 (3, 14.5)	9 (3,15)	0.3370
Single ventricle physiology (%)	19 (25.3)	11 (45.8%)	0.0572
Pre-operative enteral nutrition initiated (%)	72 (96%)	22 (88%)	0.1447
CPB time, minutes; median (range)	122 (55, 399)	131 (54, 277)	0.6595
Cross-clamp time, minutes; median (range)	69 (0, 241)	72 (0, 136)	0.7298
Deep hypothermic circulatory arrest, minutes; median (range)	0 (0, 77)	4 (0, 76)	**0.0457**
Selective cerebral perfusion, minutes; median (range)	0 (0, 115)	0 (0, 65)	0.2430
Duration of mechanical ventilation, hours; median (range)	27.8(0.1, 726.6)	82.6 (12.4, 237.8)	**0.0005**
VIS at 6 h; median (range)	8 (0, 25)	12.5 (2.5, 27)	**0.0083**
Lactate peak; median (range)	3.1 (0.9, 10.8)	5.0 (1.1, 12.6)	**0.0115**
Peak CREATININE; median (range)	0.5 (0.3, 1.1)	0.6 (0.4, 1.9)	**0.0011**
IL-6 at 6 h; median (range)	50.2 (1.3, 524.8)	61.1 (13, 296.9)	0.8819
TNFα at 6 h; median (range)	7.6 (2.7, 24.7)	7.5 (3.1, 72.3)	0.8096

*Bold values represent statistically significant values (p < 0.05)*.

### Early Enteral Feeding and Association With IFABP

Infants steadily progressed in terms of amount of enteral feeds achieved over time, with mean percent of goals kcals achieved by enteral nutrition advancing from 26% on post-op day 1, to 48% on post op day 3, to 71% on post-op day 5, to 82% on post-op day 7. On multivariable analysis, IFABP was not associated with percentage of goal enteral kcal achieved. Rather, selective cerebral perfusion time, higher Aristotle score, longer intubation time, and the need for pre-operative intubation were all independently associated with lower percentage of goal Kcals given enterally on post-op day 5, as shown in Table 5. Higher Aristotle score, longer length of intubation, need for pre-operative intubation, lower weight, and higher VIS at 24 h were independently associated with a lower percentage of goal Kcals given enterally on post-operative day 3 (Table 5).

**Table 5:** Multivariable model for predicting percent goal kcals achieved enterally.

**Independent variable**	**Goal kcals achieved enterally on POD5**			**Goal kcals achieved enterally on POD3**		
	**% Decrease per unit increase in independent variable**	**Standard error**	***p*-value**	**% Decrease per unit increase in independent variable**	**Standard error**	***p*-value**
Aristotle score	3.2	1	0.002	3	1.0	0.002
Selective cerebral perfusion (min)	0.4	0.1	0.01	–	–	–
Pre-operative intubation (yes)	14.1	6.1	0.02	15.9	6.7	0.02
Intubation time (hours)	0.1	0	0.0004	0.1	0.0	0.0007
VIS at 6 h	1.2	0.7	0.09	–	–	–
VIS at 24 h	–	–	–	1.8	0.6	0.006
Weight (kg)	–	–	–	−9.4	3.4	0.008

### Post-operative NEC and Association With IFABP

**Figure 2:**
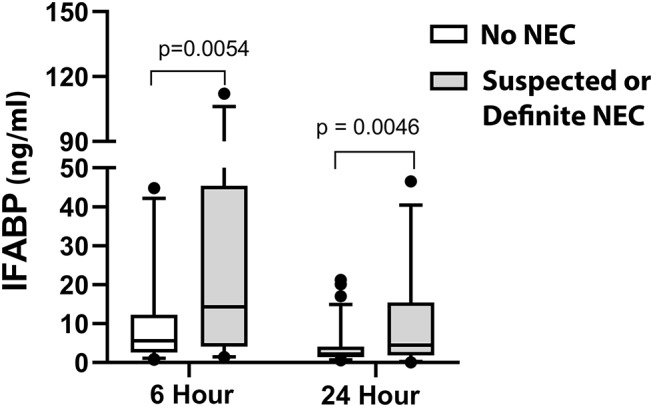
Comparison of early IFABP levels (6 and 24 h) in patients without NEC vs. with suspected or definite NEC (Modified Bell Criteria scores 1 or 2).

Of our patients, by modified Bell Staging Criteria, 75.5% (*n* = 77) were categorized as Stage 0, 15.7% (*n* = 16) were categorized as Stage 1 (suspected NEC), and 8.8% (*n* = 9) were categorized as Stage 2 (definite NEC). We did not have any patients categorized as Stage 3 (advanced NEC). None of the patients in our study had previous clinical NEC, though prior subclinical intestinal stress cannot be completely ruled out by this study design. A comparison of the distribution of IFABP levels at 6 and 24 h demonstrates a significantly higher distribution of IFABP levels at both time points in patients who subsequently developed suspected or definite NEC vs. patients who did not develop post-operative NEC (Figure 2). On multivariable analysis, IFABP and peak creatinine were independently associated with development of suspected or definite NEC, as shown in Table 6. Of note, both age and weight were initially included in our multivariable model for predicting IFABP, but they were removed in backwards selection, as they did not demonstrate a significant association with NEC when controlling for other independent variables in the model. While not significantly independently associated with development of suspected or definite NEC, VIS at 24 h improved the overall fit of the model and was therefore included.

**Table 6:** Multivariable model for predicting suspected or definite NEC (modified bell criteria scores 1 or 2).

**Independent variable**	**Percent increase in odds of NEC per unit increase in independent variable (point estimate)**	**95% wald confidence intervals**	***p*-value**
IFABP (6 h)	4% (1.04)	1.0, 1.1	0.0015
VIS (24 h)	9% (1.09)	1.0, 1.2	0.06
Creatinine (peak)	1500% (15.0)	1.4, 162.0	0.03

## Discussion

### Key Findings

In this study, we present the first evaluation of IFABP levels following cardiothoracic surgery with CPB in a specifically infant population. Additionally, we present the associations between clinical factors and pre-operative and post-operative IFABP levels. Counter to our initial hypothesis, immediate post-operative IFABP levels were not found to be associated with the percentage of goal kcals achieved enterally on post-operative day 3 or 5; rather, clinician-observable factors indicating illness severity were associated with achieved enteral kcals. We present the novel finding of an independent association in infants undergoing CPB between early enterocyte injury, as identified by circulating IFABP levels, and subsequent post-operative NEC.

### Pattern of IFABP Rise and Fall After CPB

We show that IFABP levels rise immediately after CPB, continue to rise at 6 h, and then return back to pre-operative levels by 24 h after surgery. A physiologic understanding of IFABP offers an explanation for this pattern of early rise and rapid fall. IFABP is located predominantly in the mature enterocytes in the villi as opposed to younger enterocytes in the crypts (20). These mature enterocytes are at the distal extreme of mucosal blood flow and therefore susceptible to early ischemic damage in the setting of compromised intestinal blood flow, consequently leading to a spike in systemic IFABP (20). IFABP is eliminated renally (40), resulting in a downtrend to pre-operative levels once adequate intestinal perfusion is restored. Prior studies in mixed-age pediatric cardiac surgery cohorts demonstrated a similar pattern of rise and fall in IFABP with comparable magnitudes of IFABP elevation (3, 26). Adult cardiac surgery cohorts have also shown this pattern of rise and fall in IFABP, but with smaller magnitudes of IFABP elevation (27–30, 41). This discrepancy in magnitude of IFABP elevation would be potentially explained by the differences in GI complication rates in pediatric patients vs. adult patients after cardiac surgery, with pediatric rates of just NEC ranging from 3.3 to 13% (42) and adult rates of any GI complication ranging from 0.3 to 2% (43). The early rise in IFABP is particularly notable, as early rise in IFABP and the associated intestinal barrier injury has been associated with a post-bypass inflammatory cascade (26). Normal IFABP levels are not well-defined in the infant population, and future studies would ideally include age-matched controls.

### Clinical Factors Associated With Higher Pre-operative IFABP

Higher pre-operative IFABP levels were associated with increased patient age, increased patient weight, prior initiation of pre-operative nutrition, and no prior prostaglandin use. The association of IFABP with age is both a pronounced and an interesting finding, as previously Pathan et al. (3) found no association between age or weight and IFABP levels. A potential explanation for this discrepancy is that during gestation and early infancy, IFABP levels rise as gut mass increases and IFABP accumulates. Pathan et al. (3) examined an older population (median age was 11.2 months as opposed to our median of 21.5 days) and more heterogeneous age range, and therefore potentially did not capture the initial rise in IFABP during infancy. In support of this explanation, Guthman et al. (44) has demonstrated an association between birth weight and IFABP in premature infants <33 weeks gestation age, and our data suggests that this trend may continue during early infancy as well. Examining normal IFABP levels during the infancy period would be helpful to further clarify the natural trajectory of IFABP. Likely the finding of pre-operative nutrition being associated with higher IFABP levels is collinear with the age and weight, as older patients are more likely to already have nutrition started. Another interesting finding was that prostaglandin use was associated with lower levels of IFABP, which is in contrast to a previous finding in Pathan et al. (3), who demonstrated increased IFABP in patients with ductal dependent lesions. Again, this discrepancy is most likely explained by the difference in populations: In our younger population, patients requiring prostaglandins were younger than the rest of our population and therefore the effect of their age on IFABP likely outweighed the effect of increased disease severity and decreased gut perfusion due to their ductal dependent lesions. Similarly, other indications of pre-operative disease severity (pre-operative mechanical ventilation, pre-operative inotropic support, single ventricle physiology) were also likely outweighed by the effect of age, as the more severe patients were also likely to go for their surgery at a younger age. Knowing which factors are associated with higher pre-operative IFABP is helpful in further understanding IFABP and how to properly interpret its levels.

### Clinical Factors Associated With Higher Post-operative IFABP

Higher post-operative IFABP levels were associated with longer CPB time, higher VIS, higher peak creatinine, single ventricle physiology, and duration of mechanical ventilation. Increased VIS has been prospectively shown to be associated with longer length of intubation, ICU stay, and hospital stay (35), and its correlation with higher IFABP could be due to its association with disease severity. Additionally, the physiologic state driving high VIS score (hypotension, decreased cardiac output, poor perfusion) would also likely lead to poor mesenteric blood flow, which would then be expected to cause more enterocyte injury and higher IFABP levels. A direct effect of vasoactive inotropes on mesenteric blood flow could be contributing as well, as there is evidence that vasopressin and epinephrine decrease mesenteric blood flow in both humans and (more extensively) in animal models of critical illness (45–50). Creatinine reflects perfusion of another abdominal organ, and so a physiologic state of hypoperfusion would intuitively affect both similarly. Additionally, since IFABP is renally cleared, acute kidney injury would be expected to potentiate elevated serum IFABP levels (40). Patient with single ventricle physiology have been shown to have low postprandial blood flow velocities (51), and therefore it is not surprising that higher levels of IFABP are seen in these patients. Longer CPB time's association with higher IFABP levels could potentially be due to insufficient flow during CPB, the continuous nature of flow during CPB, or it could be another surrogate for disease severity. This association has previously been documented in in adult and pediatric populations undergoing CPB (26, 41). Finally, duration of mechanical ventilation would indicate worsening disease severity and also lead to increased central pressures, venous congestion, and subsequent intestinal edema and injury. Alternatively, the intestinal injury and subsequent endotoxin leak could cause direct lung injury and cause longer duration of mechanical ventilation (52, 53). Our data confirm the previous finding in Typpo et al. (26) that CPB time and vasopressor use are associated with higher post-operative IFABP, but the remainder of the associations are novel in a pediatric population. It is of further note that none of our clinical factors found to be significantly associated with pre-operative IFABP were significantly associated with post-operative IFABP. This difference indicates that the previously discussed operative and post-operative variables outweigh preoperative variables and exemplifies the high degree of stress that cardiac surgery with CPB places on the intestine. Knowing which clinical factors are associated with higher IFABP levels can potentially help predict which infants are at higher risk for intestinal damage and its clinical sequelae.

### IFABP and Early Enteral Feeding Practices

We did not find a significant association between IFABP levels and early enteral feeding initiation/advancement, as measured by percentage of goal enteral kcal achieved by post-operative days 3 or 5. Our lack of a significant association is in contrast to the study by Typpo et al. (26) which does show an association with clinical feeding outcomes, though interestingly demonstrating that lower IFABP levels were associated with more feeding dysfunction. This discrepancy could potentially be explained by the fact that younger infants based on our data would have lower levels of IFABP based purely on age and decreased intestinal mass compared to older pediatric patients, confounding the association with feeding dysfunction.

We did show that higher Aristotle score, longer selective cerebral perfusion time, the need for pre-operative intubation, longer post-operative intubation time, and higher VIS were all associated with slower enteral feeding advances. A prior study by Alten et al., using the Pediatric Cardiac Critical Care Consortium registry to examine perioperative feeding management showed a wide variety of clinical practice surrounding feeding advancements (8), highlighting the uncertainty of perioperative feeding. The authors demonstrated a delay in initiation of post-operative feeds in hypoplastic left heart syndrome patients, who typically require longer duration of mechanical ventilation and vasoactive medications, although only univariate analysis was performed (8). Our findings on multivariable analysis are consistent with Alten's study, identifying independent associations between both surgical complexity (Aristotle scores) and post-operative clinical illness (mechanical ventilation and VIS) and delayed provision of post-operative enteral nutrition.

In both our study and the study by Alten et al., the variables found to be associated with enteral feeding initiation/advancement were known to the clinicians making the decisions regarding the speed of enteral feed advances. This finding is not surprising, as decisions to initiate and advance enteral nutrition in the early post-operative period generally hinge on observable physiologic markers and clinically visible risk factors. Whether an infant will tolerate advancing nutrition is more difficult to assess, as evidenced by the findings of Lannucci et al., who elegantly demonstrated similar clinical characteristics among infants who were initiated on post-operative enteral nutrition and did or did not subsequently proceed to develop NEC (38). Therefore development of novel biomarkers could be useful as a supplement to clinical risk factors in the assessment of readiness to feed and tolerance of enteral nutrition, particularly with the current movement toward early enteral feeding after congenital heart surgery and protocolized feeding advances (54, 55). Ideally, candidate biomarkers would provide an early signal that could be detected prior to initiation of post-operative enteral nutrition and be predictive of clinically significant feeding intolerance, especially development of NEC.

### IFABP and Post-operative NEC

In this study, we demonstrate for the first time in CHD patients an independent association between early IFABP levels and subsequent development of suspected or definite post-operative NEC. This association is consistent with prior studies in the premature neonate population (23–25, 56). Specifically, our data suggest that infants with immediate post-operative enterocyte injury, as evidenced by higher circulating IFABP at 6 h post-operatively, are at increased risk of subsequent development of suspected or definite NEC. The early appearance of IFABP in the blood (prior to the time enteral nutrition would be initiated in our post-operative infants) increases its potential value for risk stratification for timing of enteral nutrition initiation as well as speed of enteral nutrition advancement. The mechanism linking early enterocyte injury with subsequent development of NEC remains unclear. It is possible that early enterocyte injury is simply a marker for patients with an underlying predisposition for recurrent injury based on their cardiovascular or intestinal physiology, but who then have an intervening period of healing. Alternatively, it is possible that the early enterocyte injury results in an ongoing local process that does not lead to the persistent presence of circulating IFABP but remains incompletely healed. Translational animal modeling would be helpful in differentiating these two potential mechanisms. Finally, while our study was not designed to evaluate the longitudinal use of IFABP in early detection of NEC, there is growing body of evidence supporting IFABP use for this purpose in premature infants (23–25, 57), making this a promising and worthwhile target for future investigations in the CHD population.

### Limitations

Our study has several limitations. The subjects were recruited from a single institution, limiting generalizability to other centers. Additionally, while limited to infants, this study incorporated a range of congenital defects and subsequently a range of clinical severity. A large multi-institutional study would better be able to account for variances in disease process and clinical severity. The study was designed to only look at pre- and immediate post-operative IFABP, and did not look at ongoing IFABP levels, which would help better establish the temporal relationship between serum IFABP and NEC, as well as its potential utility as a point-of-care biomarker. Finally, the modified Bell Staging Criteria for NEC is imperfect in its application in the cardiac population, as it was designed primarily for the neonatal population. In particular, it is very challenging to differentiate post-operative ileus from NEC, as there is significant overlap between the radiographic and gastrointestinal findings in these two disease processes.

## Conclusion

IFABP levels rise initially following surgery with CPB in infants with CHD, then begin to fall by 24 h. IFABP levels were not associated with the clinical feeding outcome of percent of goal kcals achieved enterally on post-operative day 5. Instead, the primary predictors of clinical feeding outcomes were clinically observable factors. IFABP at 6 h was independently associated with subsequent development of suspected or definite NEC and may be useful for identifying patients at risk for development of post-operative NEC.

## Data Availability Statement

The datasets generated for this study are available on request to the corresponding author.

## Ethics Statement

The studies involving human participants were reviewed and approved by Colorado Multiple Institution Review Board. Written informed consent to participate in this study was provided by the participants' legal guardian/next of kin.

## Author Contributions

JW, PW, and JD conceived the design of the study. TU and JD performed patient recruitment. JW and TU performed data extraction. LK and JD performed IFABP analysis. JW, ST, JZ, and JD performed the data analysis and interpretation. JW and JD wrote the primary manuscript. All authors were responsible for reviewing and approving the final version of the manuscript.

## Conflict of Interest

The authors declare that the research was conducted in the absence of any commercial or financial relationships that could be construed as a potential conflict of interest.
