# Supplementary material for: Immediate Post-operative Enterocyte Injury, as Determined by Increased Circulating Intestinal Fatty Acid Binding Protein, Is Associated With Subsequent Development of Necrotizing Enterocolitis After Infant Cardiothoracic Surgery
Source: Front Pediatr. 2020 May 27;8:267. doi: 10.3389/fped.2020.00267 (PMC7267022; doi:10.3389/fped.2020.00267)
Supplement: Supplementary file 1 [file Presentation_1.PDF]

All patients are followed by by a dietitian and feeding specialists

Should we feed?

hemodynamically stable?
no history of shock or arrest?
soft abdomen, girth change <10%?
no bilious gastric output?
no NEC signs?

YES → start feeds

NO → hold feeds
- neonate or malnourished → consider PN, see guideline
- nourished children/adult → delay PN, see guideline

-single ventricle physiology
-PGE-1-dependent
-other at risk neonates

MONITOR
somatic NIRS
blood gas + lactate daily
abd. girth q 6 hrs
stool occult blood q 12 hrs

RED FLAGS
somatic NIRS <35% or trending down, trend sat. >85%
inc. lactate, inc. base excess & metabolic acidosis
trend inc. abdominal girth, trend inc. gastric residual
(+) blood in stools

contact Attending MD
stop feeding
abd. x-ray
WBC, CRP, PCT, blood cx
consider 3x antibiotics

Is pt at risk of aspiration?

- yes → small bowel feeds
- no → gastric feeds

- risk of poor perfusion
- normal GI function

TROPHIC FEEDS
0.5mL/kg/hr
hold rate for
> 12-24 hrs

CONTINUOUS FEEDS
0.5-1mL/kg/hr,*
advance by the same
rate q 4-6 hrs to goal

BOLUS FEEDS
use when ready to
transition to PO but
unable to take full

not tolerate?

Is the patient meeting his/her caloric need at maintenance fluid rate or at the current fluid order?

- yes
- no → Increase concentration by 2kcal/oz, per day, until goal calories are met. Consult Team and/or RD for concentrations > 26kcal/oz

MONITOR

*advancement guideline for infants only; for those >10kgs, suggest not initiating above 10mL/hr

P. Vichayavilas, Dec 2017

Is the patiet meeting his/her caloirc and protein needs?

Is the patient tolerating his/her feeds?
Is the patient growing adequately?

Age	BMR kcal/kg	BMR + GROWTH, kcal/kg	STANDARD RANGE FOR AGE, kcal/kg	PROTEIN, CRITICAL ILLNESS, g/kg
0-1 mos	50	95	110	2-3
1-2 mos	50	85	100	2-3
2-3 mos	50	75	95	2-3
3-6 mos	55	60	80	2-3
6-12 mos	55	60	80	2-3
1-3 yrs	60	60	80	1.5-2
4-6 yrs	50	50	75	1.5-2
7-10 yrs	40	40	70	1.5-2
11-14 yrs	30	30	60	1.5-2
15-18 yrs	30	30	50	1.5-2
Adult	25	25	25-35	1.5-2

Energy needs table above adapted and simplified from FAO/WHO/UUN Human Energy Requirements.
Note that mechanically ventilated neonates and young infants still have high growth requirements.
Infants born premature have different needs. Please consult your dietitian.
Children with cardiac failure frequently require more calories for growth than the level that is expected for age (1.2-2x higher). Continued growth monitoring is necessary to determine the adequacy of nutrition provision.

TOLERANCE
Tolerance is evidenced by absence of emesis, diarrhea, constipation; soft abdomen, with girth changes <10% from baseline. Gastric residuals are not, solely used, to assess tolerance as they do not correlate with gastric content volume, gastric emptying, reflux, vomiting or aspiration. Gastric residuals should always be used in conjunction with clinical assessment.

GROWTH
Use WHO 2006 growth velocity reference. Note that it is acceptable to gain weight at a higher rate if the patient is undernourished. Weight check: infants, daily; children, 3x/week; at least once weekly for all patients. Place newborns with birth weight ≤2kgs in isolettes. Length and OFC check, weekly for all patients ≤12 months of age.

NUTRITION LABS
If nutrition provision is adequate, prealbumin should start increasing once CRP is <2mg/dL.
A positive nitrogen balance is necessary for growth. Check UUN when the adequacy of protein provision is in question.

Nausea/Vomiting
- -Stop feeds for 1 hr, restart at same rate
- -If vomiting recurs, stop feeds for 4 hrs, then restart at 1/2 rate
- -Confirm vomiting VS spit-up
- -Consider TP Feeding
- -R/O constipation and GER
- -R/O pancreatitis with persistent vomiting/abd pain/distension

Diarrhea
- -R/O meds as contributing
- -R/O enteric pathogen
- -Check reducing substances
- -Change to isotonic or semi elemental formula
- -Persistent, stop feeds for 24 hrs
- -R/O withdrawal: systematic WAT-1 score

Constipation
- -Bowel regimen
- -Fiber containing formula for children >1 yr of age
- -Free water if volume permits

Increased abd. girth
- -Exclude constipation as contributing
- -Vent stomach
- -NPO if severe

Supplementary Figure 1 | Children's Hospital Colorado Cardiology Enteral Nutrition Guidelines.
